# Exploring the potential association and experimental validation of disrupted circadian rhythms with polycystic ovary syndrome via meta-analysis and bioinformatics: a possible pathogenic mechanism

**DOI:** 10.3389/fendo.2025.1545789

**Published:** 2025-05-22

**Authors:** Wenjia Li, Guanmei Zhang, Yue Fang, Siyi Wu, Xiaobo Song, Lushan Zhou, Lei Lei, Chenye Wang, Caifei Ding, Yixuan Wang

**Affiliations:** ^1^ Department of Reproductive Medicine, Zhejiang Chinese Medicine and Western Medicine Integrated Hospital, Hangzhou, China; ^2^ Department of Traditional Chinese Medicine, Xuhang Community Health Service Center of Jiading District, Shanghai, China; ^3^ The Second Clinical Medical College, Zhejiang Chinese Medical University, Hangzhou, China

**Keywords:** polycystic ovary syndrome, circadian rhythm, quercetin, NPAS2, BMAL1

## Abstract

**Background:**

Polycystic ovary syndrome (PCOS) has been extensively studied as a common female endocrine disease. In recent years, the relationship between circadian rhythm and PCOS has gradually drawn attention, although the precise nature of this connection remains unclear. The aim of this study was to explore further links between circadian rhythm and PCOS and to identify potential mediators of the pathogenesis of PCOS.

**Method:**

We analyzed the available data on PCOS and circadian rhythm disorders. Consequently, we identified potential transcription factors (NPAS2, INSIG1, H3F3B, SCML1) through bioinformatics and verified them in a well-established PCOS mouse model.

**Results:**

Luteinizing hormone (LH), testosterone (T), and melatonin (ML) exhibited substantial changes in the PCOS patients compared to healthy controls, with ML serving as a crucial biomarker in circadian rhythms. PCR results from ovarian tissues demonstrated altered expression of circadian core oscillator in the PCOS mouse model, with NPAS2 expression aligning with the bioinformatics analysis trend. We used quercetin (QUE) as a treatment and observed that it improved the disturbed expression of circadian core oscillations.

**Conclusion:**

Our research revealed the correlation between circadian rhythm disruptions and PCOS, identified potential targets, and provided unique insights into the pathogenesis of circadian rhythm-related PCOS. The improvement of circadian core oscillations in the QUE group offers a novel strategy for the treatment of PCOS.

## Introduction

Polycystic Ovary Syndrome (PCOS) is the most common endocrine disorder among women of reproductive age, primarily characterized by clinical hyperandrogenism or hyperandrogenemia (HA), ovulatory dysfunction (OD), and polycystic ovarian morphology (PCOM) (1). Recent epidemiological studies indicate that the global prevalence of PCOS among women ranges from 4% to 21% (2–4). Research suggests that the systemic complications of PCOS include, but are not limited to, cardiovascular diseases, abnormalities in glucose and lipid metabolism, and hypertension. Additionally, PCOS is associated with obstructive sleep apnea, endometrial cancer, and depression (5–7).

The biological clock is a biochemical mechanism that oscillates with a period of 24 hours and is synchronized with the diurnal cycle (8). The circadian rhythm is one of the primary features of an organism’s adaptation to its environment (9). In mammals, the photoneuroendocrine system, comprising the retina, suprachiasmatic nucleus (SCN) of the hypothalamus, and the pineal body, is the regulatory component responsible for generating circadian rhythms (10). Within this system, the SCN serves as the most crucial pacemaker, acting as the central biological clock of the human body, controlling many physiological and behavioral rhythms. The pineal gland is a subordinate organ regulated by the SCN and primarily secretes melatonin (ML). When the retina detects blue light intensity in the environment, it transmits light-dark signals to the pineal gland, prompting it to produce ML in darkness. ML subsequently enters the bloodstream and reaches its receptors, with its concentration in the blood exhibiting circadian rhythmic changes due to its light-dependent secretion. Evidence suggests that ML, as an endogenous timing factor, can feedback to the SCN through the bloodstream, thereby regulating the body’s circadian rhythm activities (11). Under normal circumstances, environmental light activates specialized photosensitive retinal ganglion cells (ipRGCs), which project to the SCN via the retinohypothalamic pathway. The SCN regulates its own clock to synchronize with the external environment. In addition to the SCN, clocks are also present in many other tissues, such as the liver, muscles, adipose tissue, and ovaries (12). These are collectively referred to as peripheral clocks, which the SCN can regulate through neural and endocrine mechanisms (primarily cortisol and ML) to synchronize them with the central clock. At the cellular level, molecular rhythms are regulated by transcription-translation feedback loops (TTFLs) that oscillate with an approximately 24-hour period. The positive feedback loop is driven by the heterodimerization of the circadian genes CLOCK, NPAS2, and BMAL1 within the cell nucleus, which regulate the transcription of clock-controlled genes (CCGs) encoding Period (PER) and Cryptochrome (CRY) proteins. In the circadian rhythm, PER and CRY proteins accumulate in the cytoplasm, form dimers, and translocate to the nucleus to inhibit their own transcription, thus closing the feedback loop (13). In the second major transcriptional loop, CLOCK/BMAL1 activates the transcription of nuclear receptor REV-ERBα and REV-ERBβ genes. These proteins compete with retinoic acid-related orphan receptors RORα, RORβ, and RORγ for binding sites on the BMAL1 gene (ROR response elements), providing positive (ROR) and negative (REV-ERB) transcriptional regulation (14, 15). The third feedback loop involves D-box binding protein (DBP) and nuclear factor interleukin-3 regulated protein (NFIL3, also known as E4BP4), which are regulated by CLOCK/BMAL1 and CRY1 and bind to D-box elements on circadian promoters, including those of RORα and RORβ (16–18). Synchronization of circadian rhythms with the environment is crucial for human health. Literature indicates that circadian misalignment can lead to the development of various diseases, including reproductive disorders (19, 20). For example, Li et al. reported that knockdown of clock genes in the ovaries of female mice using shRNA-mediated methods resulted in a reduced number of released oocytes (21). Similarly, Miller et al. found that CLOCK gene mutants (homozygous Clock/Clock mice) exhibited irregular estrous cycles similar to those seen in patients with PCOS (22). Studies suggest that altering the light-dark cycle in experimental animals can lead to PCOS-like changes (23, 24). Patients with PCOS often experience sleep disturbances. Researchers have observed significant alterations in serum melatonin concentrations (25) and a marked reduction in core clock genes such as CLOCK, BMAL1, and NPAS2 in peripheral blood mononuclear cells (PBMCs) of PCOS patients (26).

Disrupted circadian rhythms are closely linked to the pathogenesis of PCOS (27–29). The prevailing hypothesis suggests that alterations in circadian rhythms can trigger PCOS through mechanisms such as oxidative stress and inflammation, although the precise pathways remain unclear (30). As the reproductive lifespan of women extends, the long-term management of PCOS and fertility preservation are becoming critical areas of focus. Consequently, natural compounds with high safety profiles and minimal side effects are gaining increasing attention (31). Quercetin (QUR), a natural flavonoid widely found in Chinese herbs, fruits, leafy vegetables, and seeds, has garnered attention for its potential benefits (32). Previous studies have shown that QUR effectively lowers serum testosterone and luteinizing hormone levels in PCOS patients or animal models (33). Researchers have also observed improvements in fasting blood glucose, fasting insulin levels, and HOMA-IR with QUR treatment (34). Animal experiments indicate that QUR increases the number of preantral, antral, and preovulatory follicles, as well as corpora lutea, while reducing the number of cystic follicles (35). *In vitro* studies have demonstrated that QUR alleviates oxidative stress and apoptosis in ovarian cells (36, 37).

In order to further determine the relationship between circadian rhythm and PCOS, and to identify the potential mediators of the pathogenesis of PCOS, we conducted a comprehensive analysis integrating data on PCOS and circadian rhythm disruption. Our aim was to uncover potential associations between circadian rhythms and PCOS. Building on this foundation, we incorporated QUR as a treatment in our experiment to investigate whether QUR alters levels of circadian-related targets in PCOS mice. This approach aims to provide new insights into the treatment of PCOS.

## Materials and methods

### Meta-analysis

Search strategy: Systematic searches in PubMed, Embase, and Web of Science up to March 2024 were performed. The search terms used included the MeSH subject terms of chronobiology phenomena, chronobiology disorders, melatonin and polycystic ovary syndrome. The searching formulas are shown in **Table 1**.

**Table 1:** The searching formula of databases.

Database	Search Strategy
Pubmed	(((((((((((((((“Polycystic Ovary Syndrome”[Mesh]) OR (Ovary Syndrome, Polycystic[Title/Abstract])) OR (Syndrome, Polycystic Ovary[Title/Abstract])) OR (Stein-Leventhal Syndrome[Title/Abstract])) OR (Stein Leventhal Syndrome[Title/Abstract])) OR (Syndrome, Stein-Leventhal[Title/Abstract])) OR (Sclerocystic Ovarian Degeneration[Title/Abstract])) OR (Ovarian Degeneration, Sclerocystic[Title/Abstract])) OR (Sclerocystic Ovary Syndrome[Title/Abstract])) OR (Polycystic Ovarian Syndrome[Title/Abstract])) OR (Ovarian Syndrome, Polycystic[Title/Abstract])) OR (Polycystic Ovary Syndrome 1[Title/Abstract])) OR (Sclerocystic Ovaries[Title/Abstract])) OR (Ovary, Sclerocystic[Title/Abstract])) OR (Sclerocystic Ovary[Title/Abstract])) AND ((((((((((“Chronobiology Phenomena”[Mesh]) OR (Phenomena, Chronobiology[Title/Abstract])) OR (Chronobiology Phenomenon[Title/Abstract])) OR (Phenomenon, Chronobiology[Title/Abstract])) OR (Chronobiology Concepts[Title/Abstract])) OR (Chronobiology Concept[Title/Abstract])) OR (Concept, Chronobiology[Title/Abstract])) OR (Concepts, Chronobiology[Title/Abstract])) OR ((((((((((((“Chronobiology Disorders”[Mesh]) OR (Chronobiology Disorder[Title/Abstract])) OR (Biological Clock Disturbances[Title/Abstract])) OR (Biological Clock Disturbance[Title/Abstract])) OR (Disturbance, Biological Clock[Title/Abstract])) OR (Disturbances, Biological Clock[Title/Abstract])) OR (Psychogenic Inversion of Circadian Rhythm[Title/Abstract])) OR (Inversion Circadian Rhythm, Psychogenic[Title/Abstract])) OR (Circadian Rhythm Disorders[Title/Abstract])) OR (Circadian Rhythm Disorder[Title/Abstract])) OR (Circadian Dysregulation[Title/Abstract])) OR (Dysregulation, Circadian[Title/Abstract]))) OR (“Melatonin”[Mesh]))
Web of science	(((((((((((((((TS=(Polycystic ovary syndrome)) OR TS=(Ovary Syndrome, Polycystic)) OR TS=(Syndrome, Polycystic Ovary)) OR TS=(Stein-Leventhal Syndrome)) OR TS=(Stein Leventhal Syndrome)) OR TS=(Syndrome, Stein-Leventhal)) OR TS=(Sclerocystic Ovarian Degeneration)) OR TS=(Ovarian Degeneration, Sclerocystic)) OR TS=(Sclerocystic Ovary Syndrome)) OR TS=(Polycystic Ovarian Syndrome)) OR TS=(Ovarian Syndrome, Polycystic)) OR TS=(Polycystic Ovary Syndrome 1)) OR TS=(Sclerocystic Ovaries)) OR TS=(Ovary, Sclerocystic)) OR TS=(Sclerocystic Ovary)) AND (((((((((TS=(Chronobiology Phenomena)) OR TS=(Phenomena, Chronobiology)) OR TS=(Chronobiology Phenomenon)) OR TS=(Phenomenon, Chronobiology)) OR TS=(Chronobiology Concepts)) OR TS=(Chronobiology Concept)) OR TS=(Concept, Chronobiology)) OR TS=(Concepts, Chronobiology)) OR ((((((((((((TS=(chronobiology disorders)) OR TS=(Chronobiology Disorder)) OR TS=(Biological Clock Disturbances)) OR TS=(Biological Clock Disturbance)) OR TS=(Disturbance, Biological Clock)) OR TS=(Disturbances, Biological Clock)) OR TS=(Psychogenic Inversion of Circadian Rhythm)) OR TS=(Inversion of Circadian Rhythm, Psychogenic)) OR TS=(Circadian Rhythm Disorders)) OR TS=(Circadian Rhythm Disorder)) OR TS=(Circadian Dysregulation)) OR TS=(Dysregulation, Circadian)) OR (TS=(Melatonin)))
Embase	((Polycystic ovary syndrome or Ovary Syndrome, Polycystic or Syndrome, Polycystic Ovary or Stein-Leventhal Syndrome or Stein Leventhal Syndrome or Syndrome, Stein-Leventhal or Sclerocystic Ovarian Degeneration or Ovarian Degeneration, Sclerocystic or Sclerocystic Ovary Syndrome or Polycystic Ovarian Syndrome or Ovarian Syndrome, Polycystic or Polycystic Ovary Syndrome 1 or Sclerocystic Ovaries or Ovary, Sclerocystic or Sclerocystic Ovary) and (Chronobiology Phenomena or Phenomena, Chronobiology or Chronobiology Phenomenon or Phenomenon, Chronobiology or Chronobiology Concepts or Chronobiology Concept or Concept, Chronobiology or Concepts, Chronobiology or (chronobiology disorders or Chronobiology Disorder or Biological Clock Disturbances or Biological Clock Disturbance or Disturbance, Biological Clock or Disturbances, Biological Clock or Psychogenic Inversion of Circadian Rhythm or Inversion of Circadian Rhythm, Psychogenic or Circadian Rhythm Disorders or Circadian Rhythm Disorder or Circadian Dysregulation or Dysregulation, Circadian) or Melatonin)).af.

Inclusion criteria were as follows: (1) inclusion of articles published nationally and internationally; (2) study subjects were PCOS patients; (3) outcome indicators were melatonin, luteinizing hormone, and testosterone.

The exclusion criteria were as follows: (1) review, editorial material, meeting, conference, letter, etc.; (2) duplicate publications; (3) inability to obtain data directly or indirectly for use in the study; (4) studies with no outcome of interest.

Study selection and data extraction: Articles were independently screened by two researchers based on inclusion and exclusion criteria, and data statistics were performed using EXCEL. Extracted data included: (1) general information: first author, time of publication, country; (2) study population: number of people in the experimental and control groups, age, duration of the experiment, and intervention measures; (3) outcome of interest: statistically significant differences in melatonin, LH, and T.

Quality assessment: The quality of included studies was assessed using the risk of bias tool with RevMan 5.4. Six areas of bias were assessed (selection, performance, detection, attrition, reporting, and other biases). Two investigators independently assessed the risk of bias for each study using ‘low risk’, ‘high risk’, and ‘unclear risk’. Any disagreements were resolved by consensus or discussion with a third evaluator.

Publication bias: Publication bias was assessed using funnel plots and Begg’s and Egger’s tests, with a P value of less than 0.05 indicating publication bias.

### Bioinformatics analysis

#### Data selection and preprocessing

Two datasets related to Polycystic Ovary Syndrome (PCOS), GSE80432 and GSE193123, were downloaded from the GEO database (https://www.ncbi.nlm.nih.gov/geo/). GSE80432 served as the training set, and GSE193123 as the validation set. Genes associated with circadian rhythms (CRGs) were queried on CircaDB & MsigDB. The R package ‘SVA’ was utilized for batch correction across all datasets.

Identification of Differentially Expressed Genes (DEGs).

The R package limma was used to analyze the DEGs in the gene expression data between PCOS patients and control subjects. Genes with a P-value < 0.05 were selected as DEGs. Heatmaps and volcano plots were generated using the R packages pheatmap and ggplot2, respectively.

### Functional enrichment analysis

The R package clusterProfiler was used to perform Gene Ontology (GO) functional analysis on the DEGs, evaluating their biological processes (BP), cellular components (CC), and molecular functions (MF). Additionally, Kyoto Encyclopedia of Genes and Genomes (KEGG) pathway analysis of DEGs was conducted using the same R package. Disease Ontology (DO) enrichment analysis was performed using the R package DOSE. Gene Set Enrichment Analysis (GSEA) v4.1.0 was used to further evaluate functional enrichment of DEGs. KEGG biological pathway maps with a P-value < 0.05 were considered significantly enriched, and GSEA results with a P-value < 0.05 were also considered significant. Furthermore, the false discovery rate (FDR) adjusted P-value, defined as Q-value, was set as the critical criterion for GO and DO analyses, with Q-value < 0.05.

### Selection of hub genes

The DEGs were analyzed using the STRING database (https://string-db.org/) to construct a protein-protein interaction (PPI) network. The top five genes with the strongest correlations were further identified using the cytohubba algorithm.

### Animal experiment

#### Animals

Twenty-four female C57BL/6 mice (approximately 4 weeks old) were obtained from the Animal Center of Zhejiang Chinese Medical University (Hangzhou, China), with registration number: Certificate NO: SYXK (Zhejiang Province) 2021–0012. The mice were housed in a specific-pathogen-free (SPF) environment at a temperature of 20-26°C with a 12-hour light/dark cycle. Standard food and water were provided in the mouse cages at the Animal Research Center for one week before the experiment. All surgical procedures were approved by the Animal Ethics Committee of Zhejiang Chinese Medical University. The mice were randomly divided into three groups: the normal control group (NC), the model group (MODEL), and the quercetin group (QUE), with 8 mice in each group. After one week of adaptation feeding, all mice except for the NC group were orally gavaged with letrozole solution (1 mg/kg), while the NC mice were orally gavaged with saline for 21 days. At the same time, the QUE mice were orally administered quercetin (100 mg/kg), while the remaining mice received an equal amount of saline for 21 days. Quercetin (purity ≥97%, CAS# 117-39-5) was obtained commercially from MACKLIN Biochemical Technology Co., Ltd (Catalog No. Q817162, Shanghai, China).

### Vaginal smears

During the last 8 days of drug administration, sterile saline was used to moisten a cotton swab, which was gently inserted into the mice to collect vaginal epithelial cells. The collected cells were then smeared onto glass slides. After fixation with 95% ethanol for 10 minutes, the slides were stained with hematoxylin and eosin (H&E) for observation of changes in the morphology of vaginal epithelial cells in mice.

### Enzyme-Linked Immunosorbent Assay

Blood samples were collected via retro-orbital bleeding. After clotting at room temperature for 30 min, the blood was centrifuged (2,000 ×g, 15 min, 4°C) to isolate serum. The concentrations of testosterone (T), follicle-stimulating hormone (FSH), and luteinizing hormone (LH) in the serum were measured using commercial ELISA kits (Shanghai Enzyme-linked Biotechnology Co., Ltd., China).

### Real-Time Quantitative Polymerase Chain Reaction

TRIzol reagent (Thermo Fisher Scientific, USA) was used to extract total RNA from GC samples. The FastKing RT kit (with gDNase) was used to synthesize cDNA. qRT-PCR was performed to detect gene expression, and the primer sequences are shown in **Supplementary Table 1**.

### Statistical analysis

All data were presented as mean ± standard error of the mean (SEM). Statistical analysis was performed using SPSS 25.0 software and GraphPad Prism 9. One-way analysis of variance (ANOVA) with LSD *post hoc* analysis was used to determine the significance of differences between groups, assuming homogeneity of variance and normal distribution. Otherwise, Dunnett’s analysis was used to determine the significance of differences between groups. P-value < 0.05 was considered statistically significant.

## Results

### Meta-analysis

#### Search process and results

**Figure 1:**
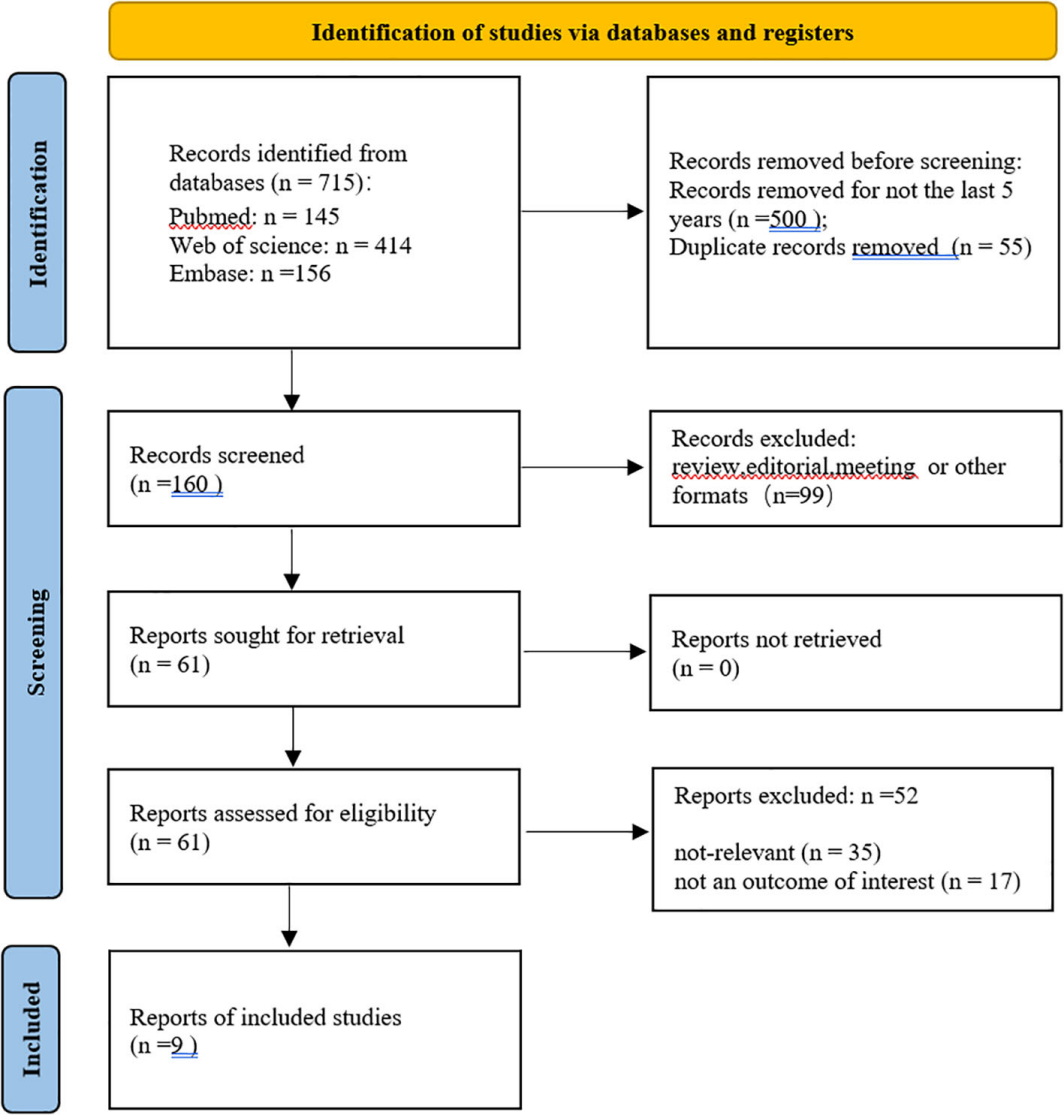
Search process and results.

**Figure 2:**
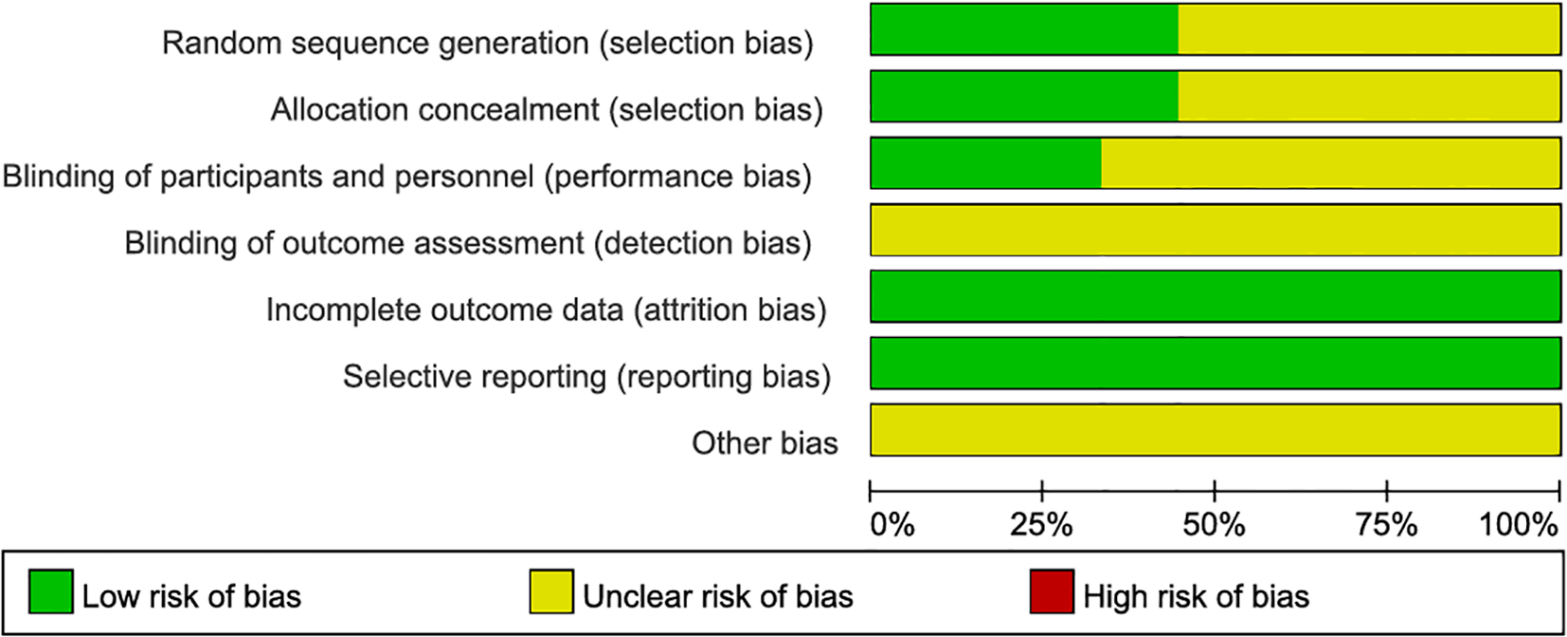
Quality assessment.

A total of 715 studies were searched, 500 studies that were not from the last 5 years were excluded, and 55 duplicates were excluded. After further reading, 99 non-experimental studies were excluded, 35 studies were excluded after screening titles and abstracts, 17 were excluded for unavailability of important information, and finally, 9 studies were obtained for inclusion in the study (**Figure 1**, **Table 2**). The quality evaluation is shown in **Figure 2**.

**Table 2:** Characteristics of the included studies.

Studies	Research object	Sample Size (Intervention/Control group)	Age(years)	Duration	Intervention Measure		Outcome
					Intervention	Control	
Jamilian, M.(2019,Iran) (38)	PCOS patients	56 (28/28)	18-40	12 weeks	melatonin	placebo	Serum total testosterone
Akter, S, (2023, Bangladesh) (39)	PCOS patients	74 (40/34)	18-35	8 weeks	melatonin	no	Serum testosterone
Alizadeh, M.(2021, Iran) (40)	PCOS patients	41 (21/20)	18-40	8 weeks	melatonin	placebo	Testosterone
Li,H.(2022,China) (25)	PCOS and non-PCOS women	71 (35/36)	25-35	/	/	/	Intrafollicular Melatonin, Testosterone
Barrea, L.(2023, Italy) (29)	PCOS and non-PCOS women	224 (112/112)	/	/	/	/	Testosterone
Yu, K.(2019,China) (41)	PCOS and non-PCOS women	30 (15/15)	25-35	/	/	/	Intrafollicular Melatonin, Testosterone, LH
Lim, A. J. R.(2019, Singapore) (42)	PCOS and non-PCOS women	163 (41/122)	21-45	/	/	/	Serum melatonin, Testosterone , LH
Simon, S. L.(2019, United States) (20)	PCOS and non-PCOS women	92 (59/33)	12-21	/	/	/	Salivary-melatonin, Testosterone
Abduljabbar H, A.(2019, Iraqi) (43)	PCOS and non-PCOS women	90 (60/30)	15-35	/	/	/	Serum melatonin

### Forest plot and funnel plot

**Figure 3:**
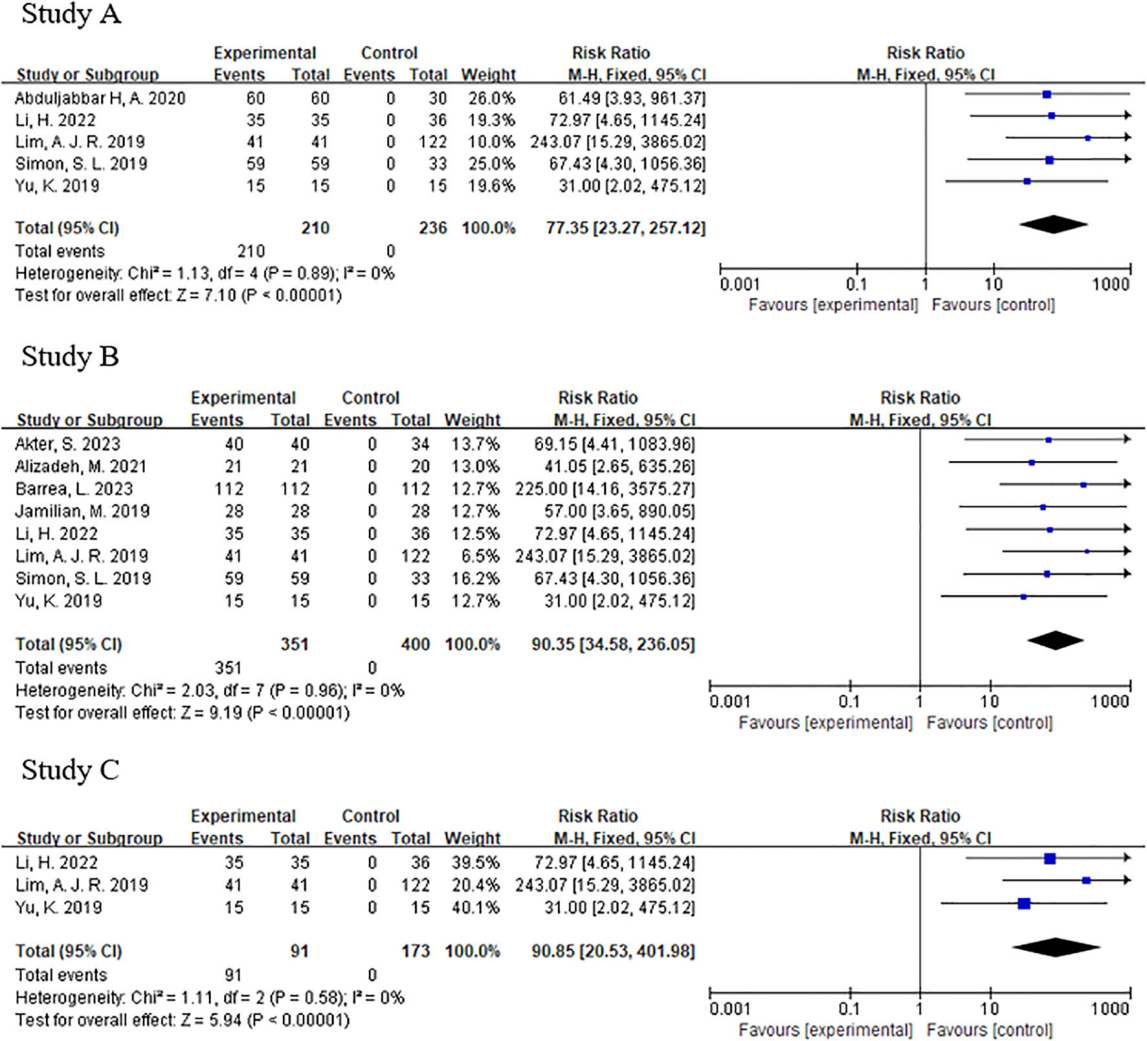
**(A)** Forest plot of melatonin between pcos and non-pcos patients. **(B)** Forest plot of testosterone between pcos and non-pcos patients. **(C)** Forest plot of luteinizing hormone between pcos and non-pcos patients.

**Figure 4:**
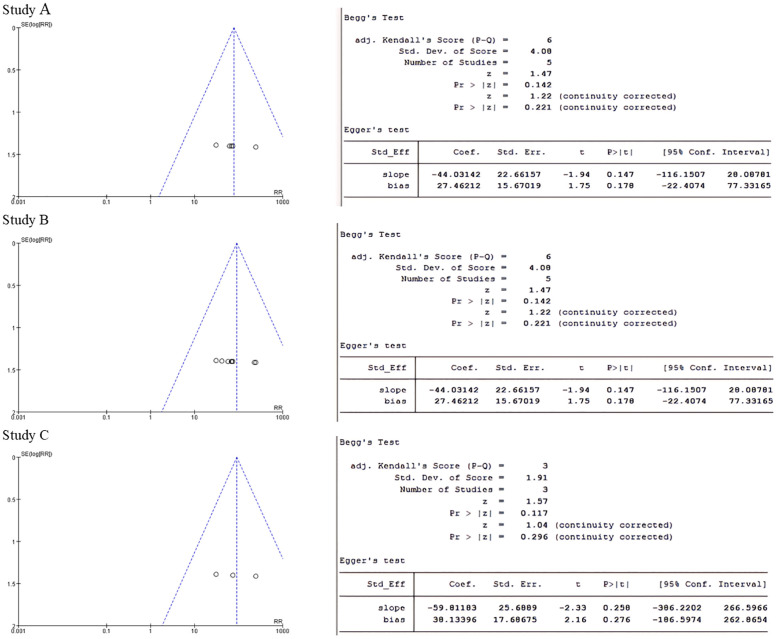
**(A)** Funnel plot of melatonin group and Begg's test and Egger's test for p-value. **(B)** Testosterone group funnel plot and Begg's test and Egger's test to test p-values. **(C)** Luteinising hormone group funnel plot and Begg's test and Egger's test for p-values.

The forest plot showed significantly difference in melatonin level, testosterone level and luteinizing hormone level between PCOS and non-PCOS patients (**Figure 3**). The p-values for both Begg’s test and Egger’s test were greater than 0.05, indicating that none of them had publication bias (**Figure 4**).

### Relationship between circadian rhythms and PCOS

**Figure 5:**
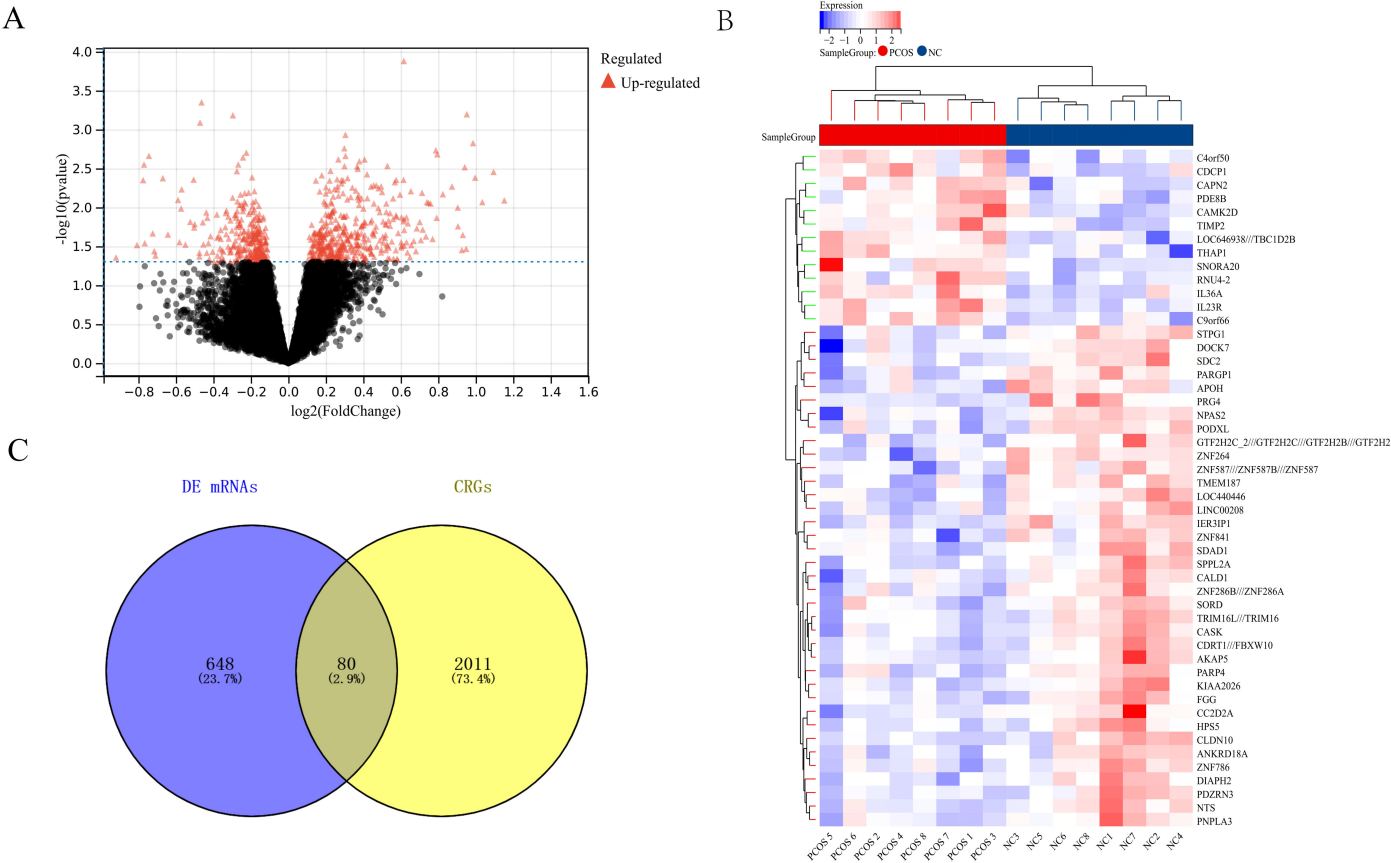
Relationship between Circadian Rhythms and PCOS **(A)** Volcano plot showing differentially expressed genes between the PCOS and NC groups. **(B)** Specific expression characteristics of differentially expressed genes in the PCOS and NC groups. **(C)** Relationship between differentially expressed genes and circadian rhythm-related genes (CRGs).

We analyzed the training dataset GSE80432. Compared to the NC group, a total of 728 DEGs were identified in the PCOS group (**Figure 5A**). The heatmap (**Figure 5B**) displays the differential expression of these DEGs between the PCOS and NC groups. To explore the relationship between PCOS and circadian rhythms, we further analyzed the DEGs with circadian rhythm-related genes (CRGs) and identified 80 common genes (**Figure 5C**). This result suggests the potential involvement of circadian rhythms in PCOS.

### Enrichment analysis

**Figure 6:**
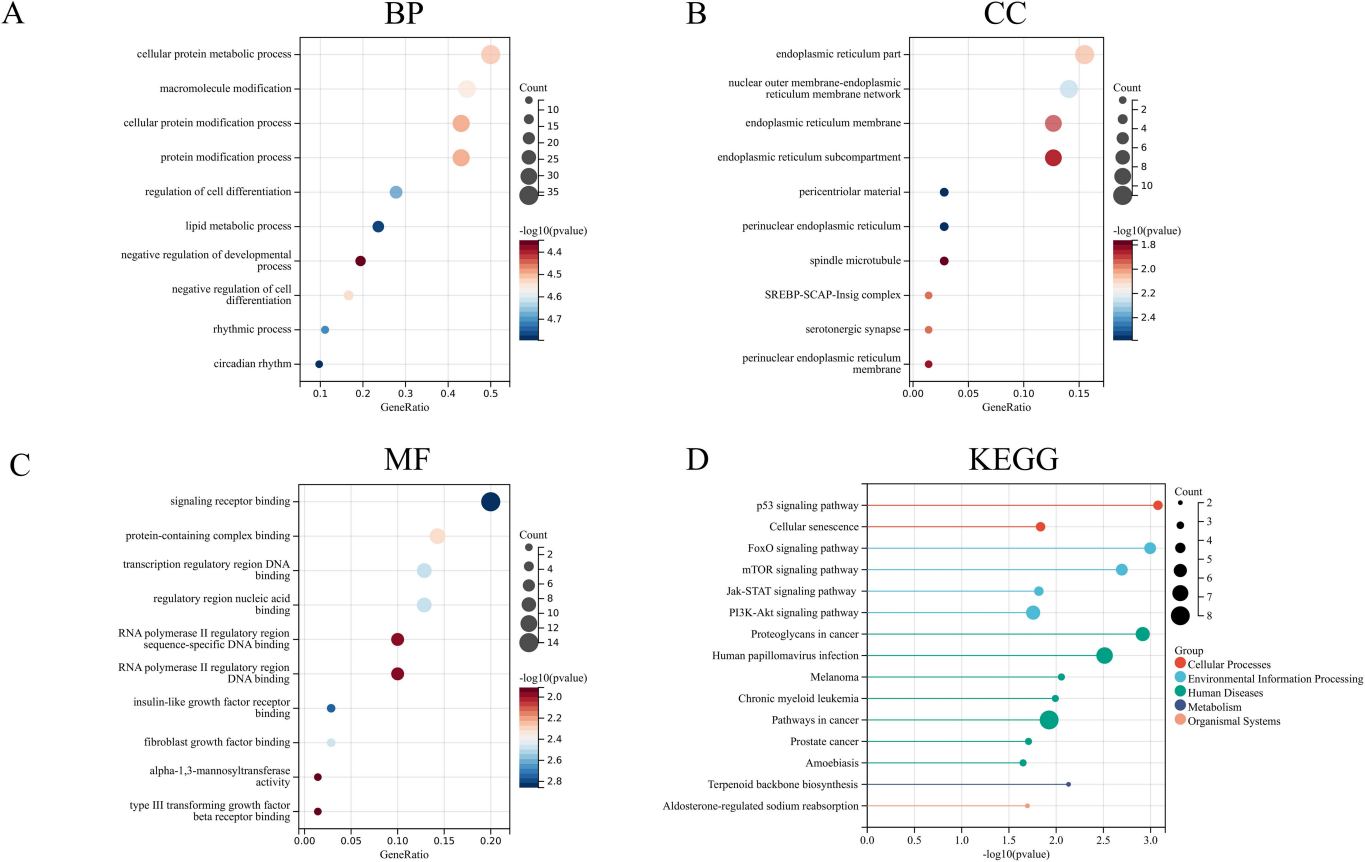
GO enrichment analysis and KEGG pathway analysis **(A)** Biological process. **(B)** Cellular component. **(C)** Molecular function. **(D)** KEGG pathway.

Enrichment analysis, including GO and KEGG pathway analysis, was performed on the selected 80 genes (**Figure 6**). The GO enrichment analysis revealed that the potential targets of these genes were mainly associated with the endoplasmic reticulum, the endoplasmic reticulum membrane, and the endoplasmic reticulum subcompartment for cellular components. The molecular functions included RNA polymerase II regulatory region sequence-specific DNA binding and RNA polymerase II regulatory region DNA binding. The biological processes were primarily related to cellular protein metabolic processes, macromolecule modification, cellular protein modification processes, and protein modification processes. The KEGG pathway analysis included pathways such as the p53 signaling pathway, mTOR pathway, FoxO pathway, and human papillomavirus infection. Notably, at the cellular component level, these genes were closely related to the endoplasmic reticulum, providing valuable insights for our subsequent research.

### Hub genes

**Figure 7:**
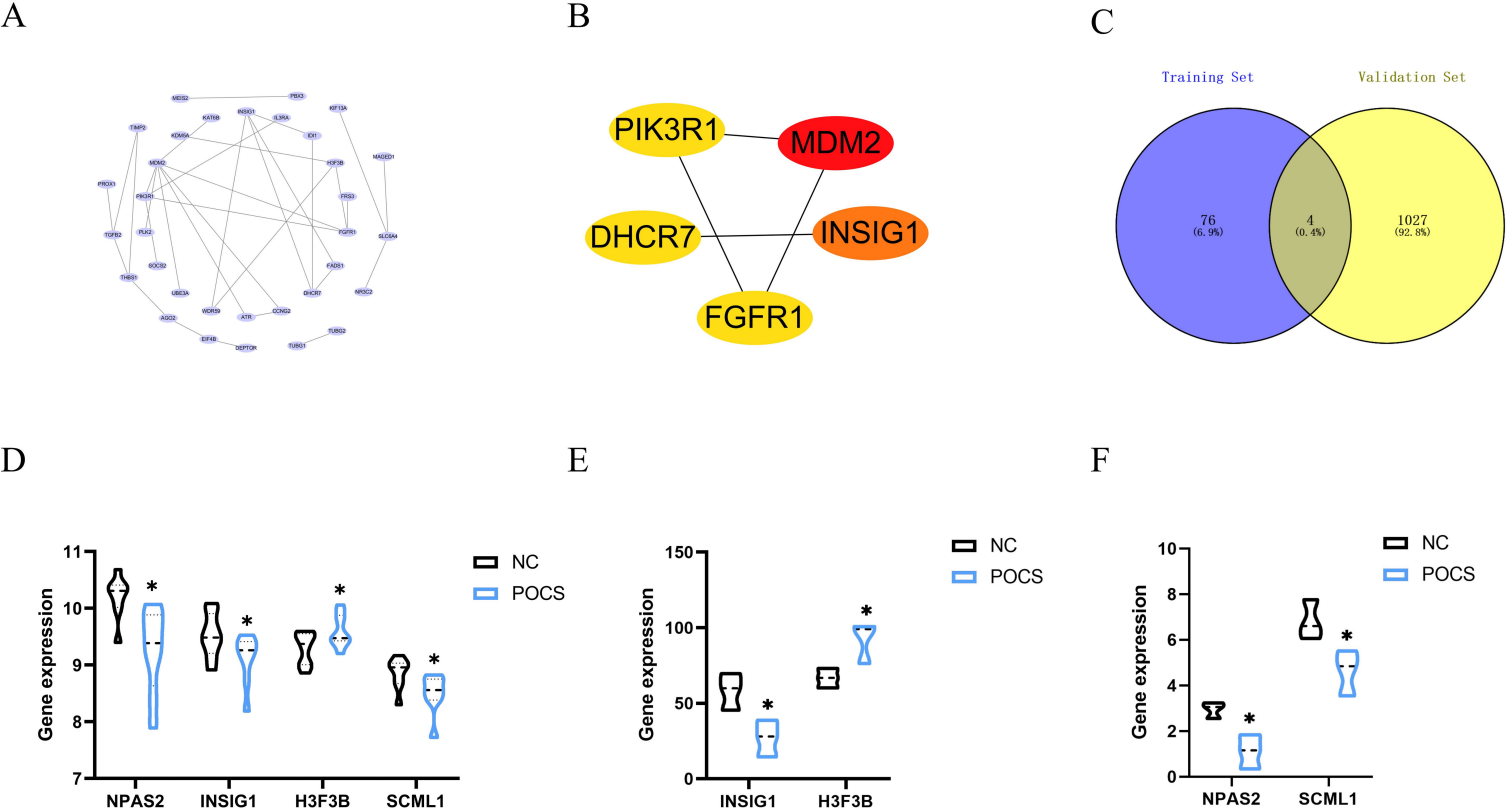
Hub genes **(A)** Protein-protein interaction network among the 80 genes. **(B)** Top five genes with the strongest correlations identified using the cytohubba algorithm. **(C)** Intersection of the 80 genes with the validation dataset. **(D)** Specific expression of intersected genes in the training dataset. **(E, F)** Specific expression of intersected genes in the validation dataset. * P<0.05.

To further explore the relationship among these 80 genes, we constructed a protein-protein interaction network using the STRING database (**Figure 7A**) and identified the top five genes with the strongest correlations using the cytohubba algorithm (**Figure 7B**). These genes are MDM2, INSIG1, FGFR1, DHCR7, and PIK3R1. Additionally, we took the intersection of the 80 genes with the differentially expressed genes in the validation dataset GSE193123, leading to four common genes (**Figure 7C**): NPAS2, INSIG1, H3F3B, and SCML1. These four genes exhibited the same change trend in expression between the training and validation datasets (**Figures 7D–F**). This result provides potential target genes through which circadian rhythms may act on PCOS.

### Animal validation

**Figure 8:**
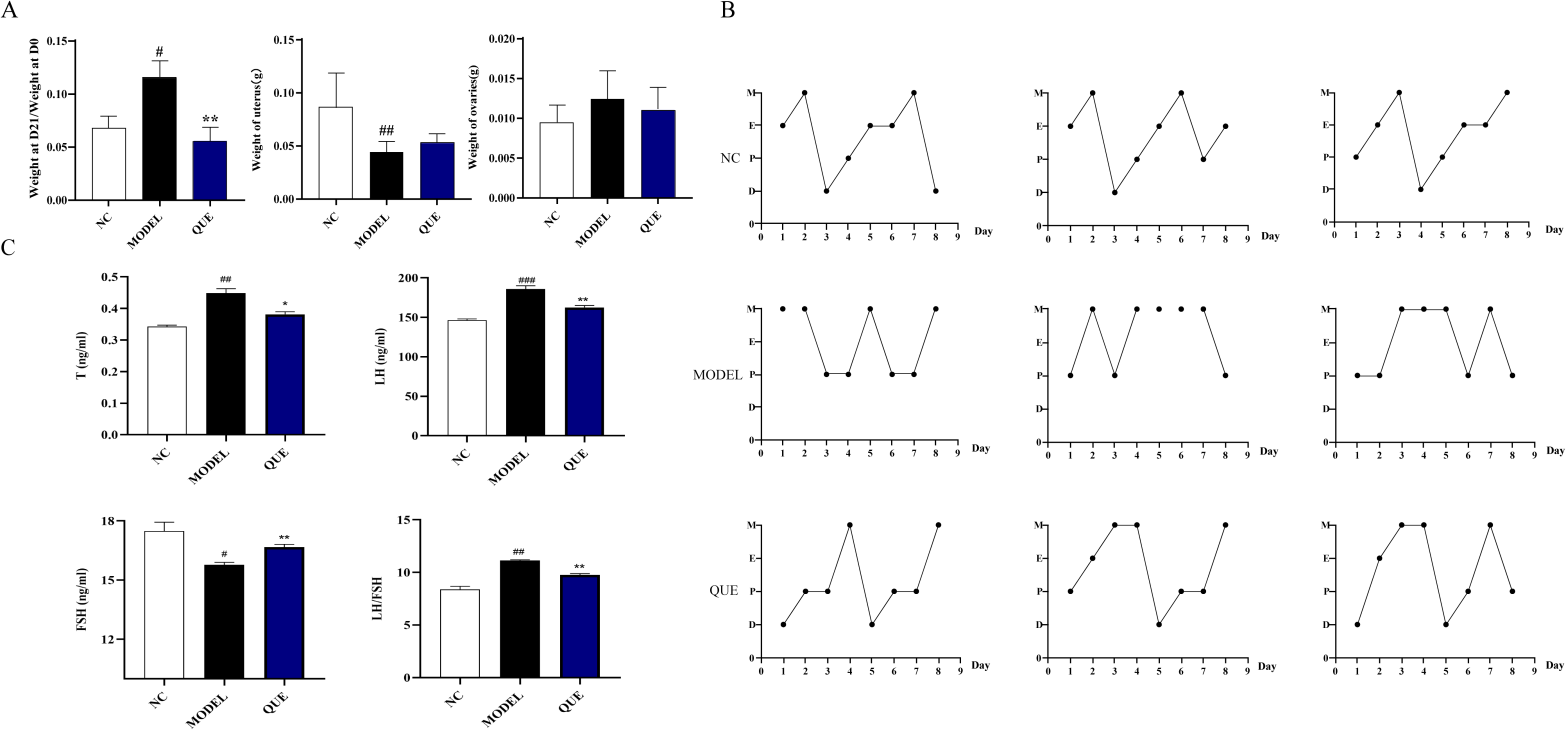
PCOS mouse model. **(A)** Day 21 body weight change, uterine weight, and ovarian weight. **(B)** Estrous cycle. **(C)** Reproductive hormone levels determined by ELISA. All data are presented as mean ± standard error of the mean (SEM) (n≥3). ###P<0.001, ##P<0.01, and #P<0.05 vs. NC group; **P<0.01, and *P<0.05 vs. Model group.

**Figure 9:**
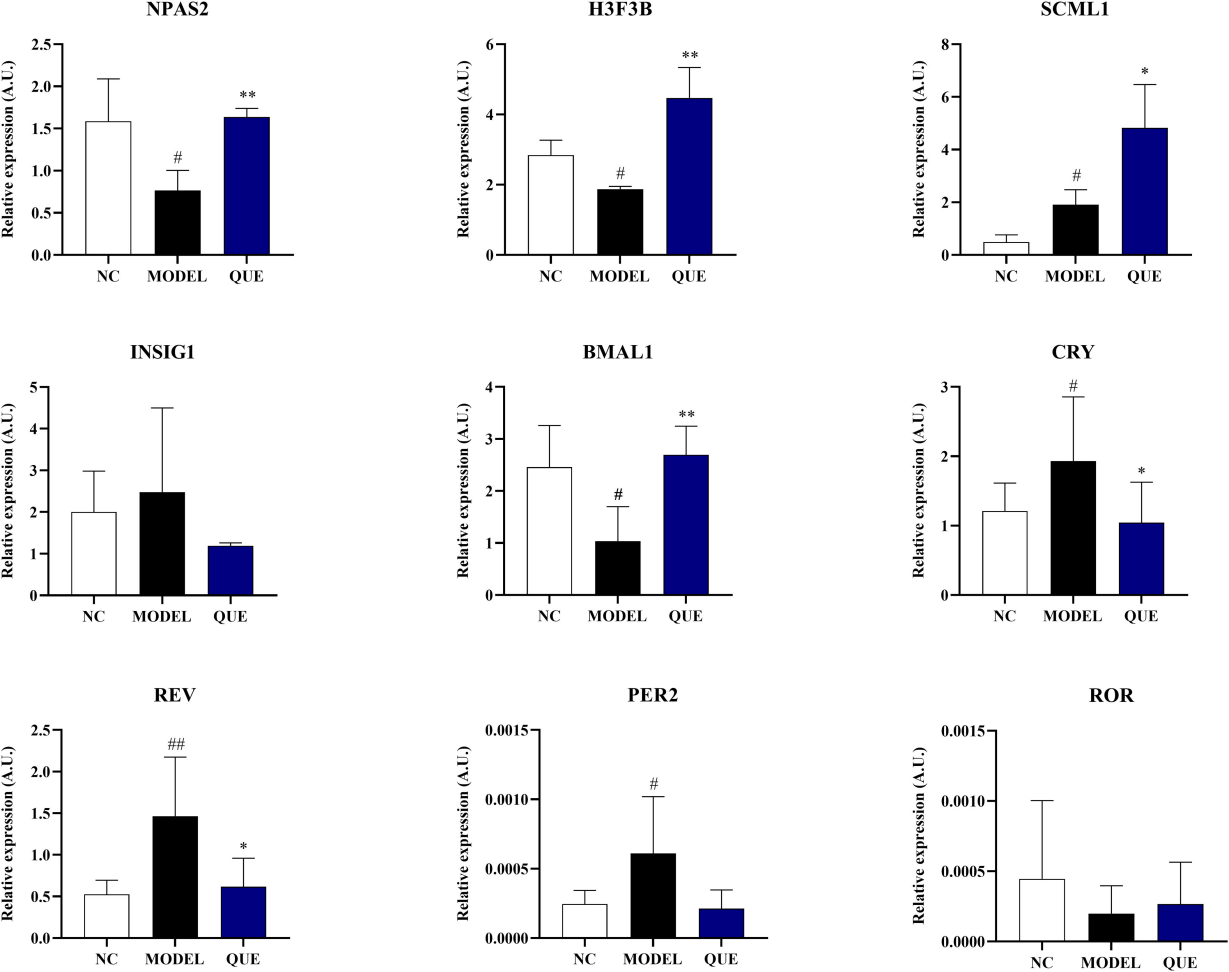
Data validation in the mouse model. All data are presented as mean ± standard error of the mean (SEM) (n≥3). ##P<0.01, and #P<0.05 vs. NC group; **P<0.01, and *P<0.05 vs. Model group.

By administering letrozole for 21 days, we successfully established the PCOS mouse model. Compared to the NC group mice, the MODEL group mice showed significant changes in body weight and uterine weight on day 21 (**Figure 8A**). The estrous cycle also displayed disruptions (**Figure 8B**). We measured reproductive hormones in mice serum using ELISA and found that, compared with NC group mice, MODEL group exhibited a significant increase in T levels, accompanied by a decrease in FSH levels and an increase in LH levels (**Figure 8C**). Concurrently, the QUE group of mice showed significant amelioration of these changes following the administration of quercetin. We also employed qRT-PCR to detect the expression of circadian core oscillator (BMAL1, NPAS2, ROR, CRY, REV, PER2) and potential transcription factors (NPAS2, INSIG1, H3F3B, SCML1) in the ovarian tissues of the mice (**Figure 9**).

The results demonstrated that the expression of circadian core oscillator gene BMAL1 in the MODEL group was significantly lower than that in the NC group, and the expression of ROR also exhibited a downward trend without statistical discrepancy. Consistently, the expression of oscillator negative feedback genes (CRY, REV, and PER2) was increased. These indicate that the circadian core oscillator’s function is compromised in PCOS mice. We then further validated the expression of previously identified transcription factors. The expression level of NPAS2 showed a similar trend to the bioinformatics analysis, whereas the expression of SCML1 and H3F3B was completely opposite. In our study, we used quercetin as a pharmacological intervention and observed that it reversed the expression of circadian core oscillators (BMAL1, NPAS2, CRY, REV, PER2) and potential transcription factors (NPAS2, H3F3B). However, the expression of SCML1 was significantly increased in the ovaries of the quercetin treatment group.

## Discussion

As of now, the specific pathogenesis of polycystic ovary syndrome still remains unclear. However, both environmental and genetic factors clearly play crucial roles. The prevailing consensus suggests a strong correlation between circadian rhythm disruption and the pathophysiology of PCOS, with specific mechanisms remaining under exploration (44–46). This study revealed significant variations in LH, T, and ML levels between the polycystic ovarian syndrome cohort and the healthy control group by meta-analysis. Given that ML functions as a vital biomarker of circadian rhythms and reflects the level of individual circadian rhythm disruption. Thus, we infer a substantial link between circadian rhythm disruption and polycystic ovary syndrome. To further examine this association, we performed bioinformatics analysis to identify potential transcription factors (NPAS2, INSIG1, H3F3B, SCML1) and verified their expression levels in both the training and validation datasets. Given that current research demonstrates the reciprocal impact of androgens and circadian rhythm disruptions (47), both of which contribute to the pathophysiology of PCOS (24), we opted to utilize letrozole to simulate hyperandrogenism. Subsequently, we examined the alterations in core clock genes in the ovaries of this mouse model, validated the expression of previously identified transcription factors, and evaluated the *in vivo* effects of quercetin, which produced intriguing and fascinating findings.

Our study described the dynamic regulatory network of four transcription factors—NPAS2, INSIG1, H3F3B, and SCML1—in the pathological progression of polycystic ovary syndrome (PCOS) and their intricate associations with circadian disorders by integrating bioinformatics analysis and animal experimental validation. Our bioinformatics analysis indicated a significant down-regulation of NPAS2, INSIG1, and SCML1, whereas H3F3B exhibited up-regulation, suggesting that these genes may contribute to the pathogenesis of PCOS through the circadian and metabolic regulatory network. NPAS2 is a vital circadian gene that dimerizes with BMAL1 to modulate the transcription of downstream rhythmic genes, including Period (PER) and Cryptochrome (CRY) genes. Its function in the suprachiasmatic nucleus (SCN) parallels that of the CLOCK gene, which can be compensated for NPAS2 in its absence to maintain the cyclic oscillations of the biological clock (48). The current study demonstrated a coordinated down-regulation of NPAS2 expression in the mouse model, alongside a reduction in ovarian BMAL1 expression, consistent with the notable decrease of NPAS2 levels in the peripheral blood of PCOS patients reported (26), as well as the diminished expression of BMAL1 in ovarian granulosa cells (46). Accompanied by an increase in the negative feedback genes, PER2 and CRY (26), these indicate that the circadian core oscillator’s function is compromised in PCOS, where the BMAL1/CLOCK or BMAL1/NPAS2 dimer plays a significant role. Metabolic abnormalities are clinical characteristics of PCOS, such as insulin resistance, dysregulated lipid metabolism, and altered cholesterol metabolism, which may also be present in patients with circadian rhythm disorders (49). Research by Li et al. showed that sustained darkness decreased BMAL1 expression in rat liver and adipose tissue while facilitating insulin resistance via GLUT4 (44). Furthermore, the negatively regulated gene BMAL1, REV-ERB, plays a role in the regulation of metabolic processes, including insulin resistance and lipid metabolism problems (50, 51), and exerts an inhibitory influence on granulosa cell apoptosis in PCOS (52). This indicates that the circadian core oscillator plays a role in the pathophysiology of PCOS by affecting the metabolic regulatory network. INSIG1 is a protein located in the endoplasmic reticulum that primarily participates in the negative feedback regulation of cholesterol production. Increased intracellular cholesterol levels prompt INSIG1 to bind to HMG-CoA reductase, inhibiting its activity and also suppressing the activity of upstream SREBP, which ultimately diminishes cholesterol synthesis (53, 54). In the PPI network of this study, DHCR7 exhibits a strong correlation with INSIG1, presumably due to their mutual participation in cholesterol synthesis. INSIG1 primarily functions by negative regulation of this process, while DHCR7 directly catalyzes the conversion of 7-dehydrocholesterol (7-DHC) to cholesterol (55, 56) and participates in vitamin D synthesis (57). While no research has thoroughly clarified the regulation of INSIG1 or DHCR7 by BMAL1/NPAS2, it has been demonstrated that BMAL1 modulates the expression of the cholesterol-synthesizing rate-limiting enzyme HMG-CoA reductase (HMGCR) gene (58, 59) and that BMAL1 significantly contributes to the upstream SREBP of INSIG1 via SIRTs (60, 61) and steroid biosynthesis in human luteinized granulosa cells (62). These findings suggest the need to investigate BMAL1/NPAS2 in the context of female granulosa cell steroid metabolism, starting with INSIG1 or DHCR7. In the animal validation portion of the current investigation, it is noteworthy that while NPAS2 expression in the ovaries of the PCOS model mice showed substantial suppression, INSIG1 did not exhibit the expected decrease in expression. This indicates that cholesterol metabolism may not be the primary mechanism by which BMAL1/NPAS2 affects the etiology of PCOS. However, it does not rule out the potential association with compensatory up-regulation, nor the limitations of this mouse model, which is predominantly characterized by hyperandrogenism, as well as the interspecies variability in lipid metabolism regulation, both of which require further research.

SCML1 is a meiosis-specific protein closely associated with the progression of meiosis, expressed exclusively in spermatocytes at the L1 stage, and functions as a vital marker gene in human spermatogenesis (63). H3F3B is a histone variation commonly associated with DNA damage repair, chromatin remodeling, and the regulation of gene expression. The expression of both in biosynthesis analysis indicates the presence of DNA damage and chromatin remodeling in PCOS, consistent with observations from certain studies (64, 65). The expression of these two variations in the model mouse ovary indicates DNA damage and chromatin remodeling in PCOS, corroborating findings from specific research (64, 66) and supporting the theoretical framework concerning granulosa cell apoptosis and DNA damage in PCOS patients. Conversely, the model mouse ovary exhibits opposing expression, possibly linked to the extensive follicular atresia and the failure of compensatory repair mechanisms. Given that the animal validation experiments utilized the whole ovary and the data for the bioinformatics analysis were derived from human ovarian granulosa cells, further validation in granulosa cells may be pursued in the future. The robust MDM2, FGFR1, and PIK3R1 protein interaction network is strongly associated with granulosa cell death in PCOS. Current research suggests that the P53 and PI3K/AKT pathways are participating in granulosa cell apoptosis, insulin resistance, and lipometabolic abnormalities in PCOS. Furthermore, the study by Wang W et al. suggests that BMAL1 may influence hormone synthesis and the apoptosis of porcine granulosa cells via the PI3K/AKT pathway (67). Taken together, these data suggest that circadian genes in PCOS peripheral ovarian granulosa cells function within a multifaceted dynamic network, influencing numerous features such as granulosa cell proliferation, DNA damage, lipid metabolism abnormalities, and insulin resistance. Sterol metabolism and cell cycle perspectives should be considered in further studies of the relationship between circadian rhythms and PCOS.

Quercetin is a representative flavonoid widely found in herbs, fruits, and vegetables, and it has been recognized as an active core component of Traditional Chinese Medicine (TCM) in several TCM compounding studies, and it was also a core active component of the Cuscuta-Danshen pair, a featured drug pair in the PCOS treatment in our previous study (68). As one of the most popular dietary flavonoids, quercetin has strong antioxidant effects due to the presence of double bonds and carboxyl groups in its structure (69) and has shown strong pharmacological activities in various studies compared to other flavonoids, with biological functions related to antioxidant, anti-inflammatory, antihypertensive, and anti-diabetic properties. Quercetin has demonstrated its multi-targeted regulatory effects in gynecological diseases such as PCOS, premature ovary failure (POF), endometriosis (EM), ovarian cancer (OC), and cervical cancer (CC), especially in PCOS. On the one hand, quercetin down-regulates the expression of aromatase due to its E2-like structure and inhibits androgen biosynthesis through the PI3K pathway, as well as inhibits the expression of androgen receptor (AR) and activates PI3K/A2K/AR to reduce androgen levels. On the other hand, quercetin activates the PI3K/Akt pathway and the AMPK pathway to alleviate insulin resistance and lipid metabolism disorders. Furthermore, quercetin exerts its powerful anti-inflammatory and antioxidant effects in PCOS. In two randomized, controlled, double-blind clinical trials (70, 71), a daily dose of 1000 mg of quercetin for 12 weeks reduced hormonal parameters and HOMA-IR levels in PCOS patients. As a phytochemical, the role of quercetin in the regulation of circadian rhythms is increasingly recognized (72, 73). In our study, we used quercetin as a pharmacological intervention in the PCOS model to observe its effects. We were pleased to see that quercetin had an ameliorative effect on the gene expression of the circadian core oscillators (BMAL1, NPAS2, CRY, PER2, and REV-ERB), promoting the expression of the BMAL1 gene and deregulating the negative feedback on the expression of PER2/CRY and REV-ERB. Although a more comprehensive and in-depth study is required, this phenomenon at least suggests that the therapeutic effect of quercetin on PCOS can be interpreted from the perspective of circadian rhythms, which establish a therapeutic regimen for PCOS patients at work, whose circadian rhythms are inevitably disrupted. Moreover, since the gene expressions of both H3F3B and SCML1 were significantly increased in the quercetin treatment group, it is a new perspective to explore the ameliorative effect of quercetin on circadian disorders in PCOS. Since the expression of SCML1 was significantly increased in the ovaries of the quercetin treatment group, we believe that the effects of quercetin on oocytes should be further explored, and perhaps striking discoveries will come to the surface.

Our investigation presents a fresh perspective by discovering and verifying possible transcription factors linked to circadian rhythm disruption in the pathogenesis of PCOS, as well as evaluating the therapeutic effects of quercetin in a PCOS model; nevertheless, our shortcomings are also obvious. Initially, we selected the letrozole PCOS mouse model, primarily distinguished by hyperandrogenism and a less pronounced metabolic phenotype compared to the letrozole-high-fat diet model. Its effects may not be entirely apparent in the animal validation phase, as the regulatory functions of the circadian core oscillator predominantly influence glucose and lipid metabolism. Secondly, we only confirmed the expression of the circadian core oscillator and predicted transcription factors we found earlier in the ovary of the PCOS model, whereas the validation pertaining to glycolipid metabolism was deferred due to insufficient samples. Furthermore, since the validation was performed at the ovarian level instead of the granulosa cell level, the explanation of some certain results was ambiguous. Future investigations could delve deeper into the specific regulatory mechanisms of rhythmic genes on glycolipid metabolism in the hypothalamic rhythm center or across various cell types in PCOS ovaries.

## Conclusion

**Figure 10:**
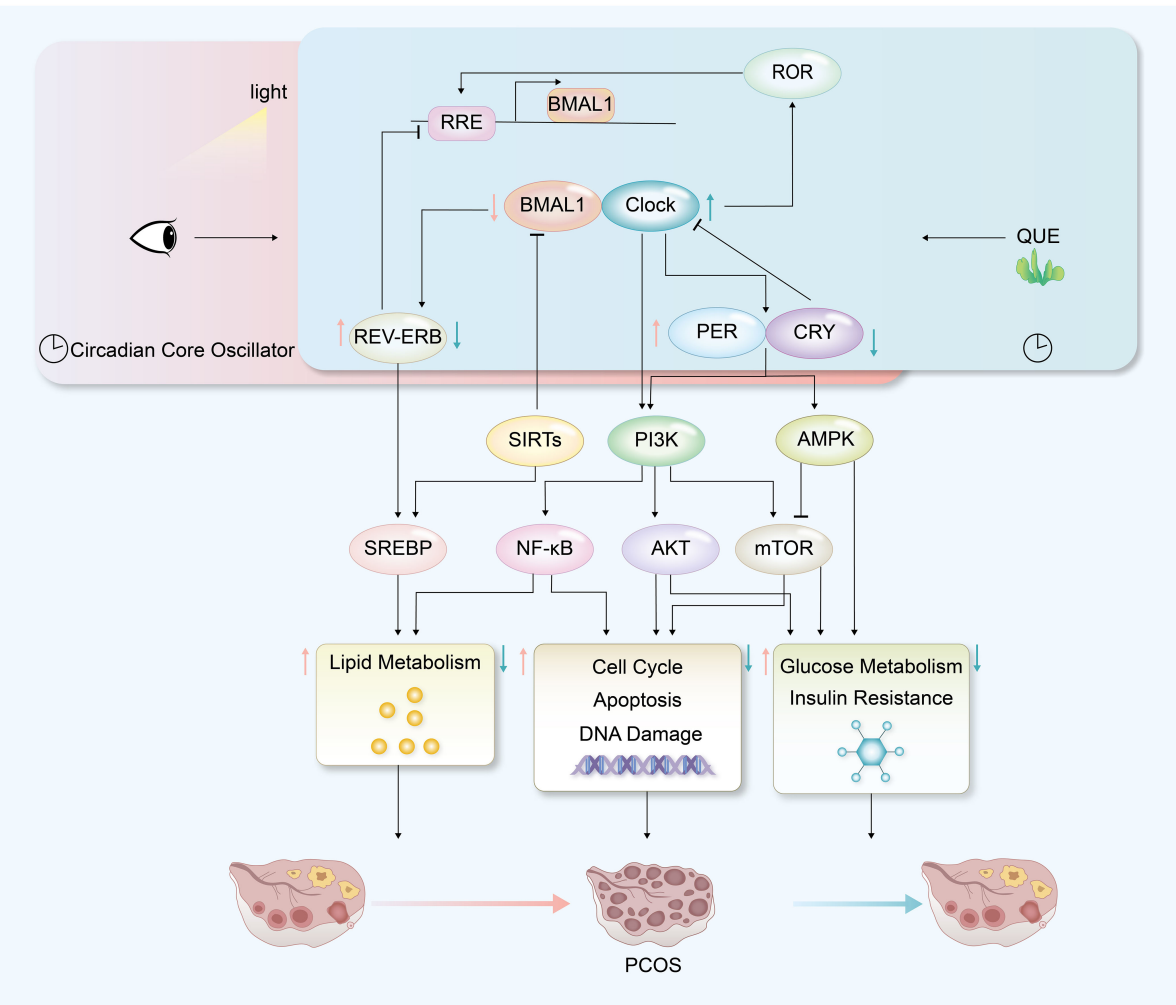
Hypothetical mechanism diagram of PCOS with circadian disruption and quercetin treatment.

Our study conducted a meta-analysis based on existing data and successfully identified a correlation between disrupted circadian rhythms and PCOS. Further bioinformatics analysis led to the identification of potential transcription factors, which were validated in mouse models. This research actively explored and speculated on possible molecular mechanisms linking circadian rhythm disruption to PCOS (**Figure 10**), which could facilitate the development of circadian-guided therapeutic strategies for polycystic ovary syndrome. In addition, the involvement of quercetin treatment in animal experiments provides new insights for future PCOS treatments.

## Data Availability

The datasets presented in this study can be found in online repositories. The names of the repository/repositories and accession number(s) can be found in the article/**Supplementary Material**.
