# Supplementary figures and images for: Exploring the potential association and experimental validation of disrupted circadian rhythms with polycystic ovary syndrome via meta-analysis and bioinformatics: a possible pathogenic mechanism
Source: Front Endocrinol (Lausanne). 2025 May 22;16:1545789. doi: 10.3389/fendo.2025.1545789 (PMC12137095; doi:10.3389/fendo.2025.1545789)

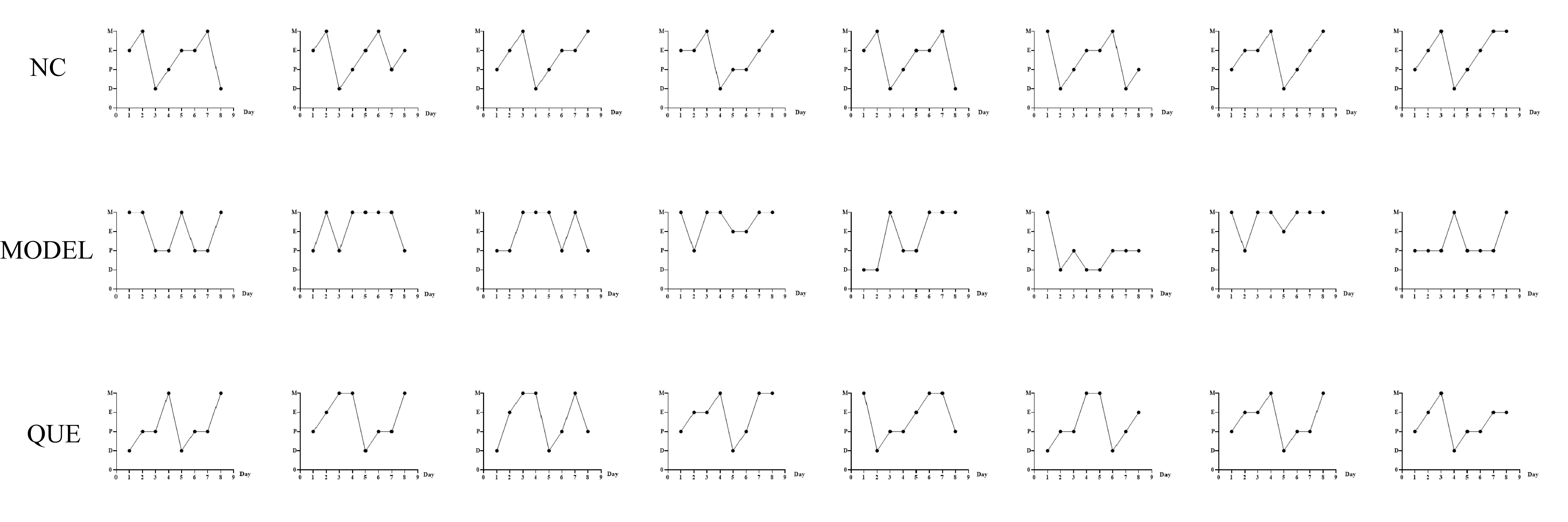

Supplement: Supplementary file 1 [file Image1.tif]

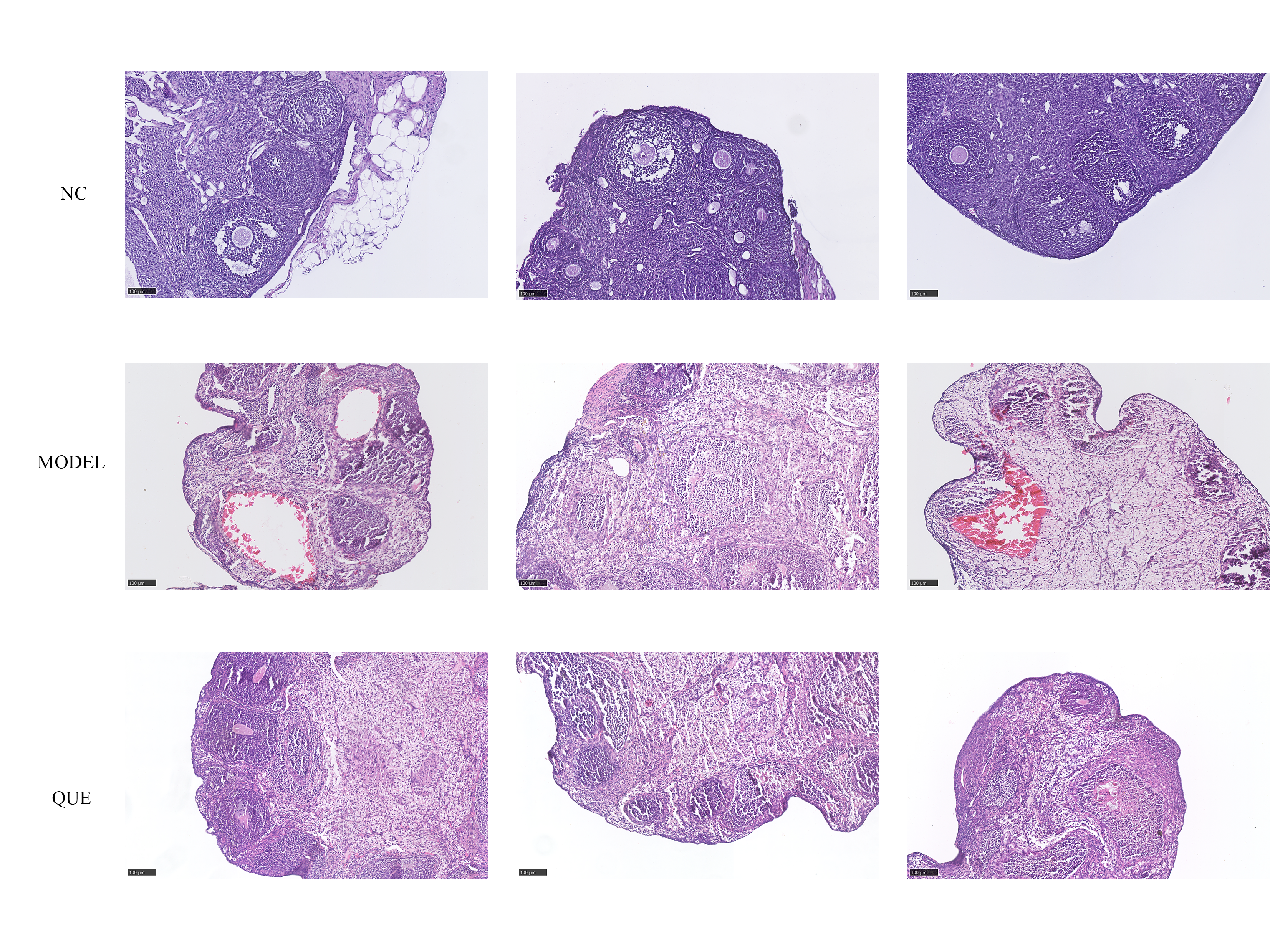

Supplement: Supplementary file 2 [file Image2.tif]
