# Supplementary material for: Exploring the potential association and experimental validation of disrupted circadian rhythms with polycystic ovary syndrome via meta-analysis and bioinformatics: a possible pathogenic mechanism
Source: Front Endocrinol (Lausanne). 2025 May 22;16:1545789. doi: 10.3389/fendo.2025.1545789 (PMC12137095; doi:10.3389/fendo.2025.1545789)
Supplement: Supplementary file 3 [file Table1.docx]

Supplementary Table 1.

| Primer name | serial number |
| --- | --- |
| M-NPAS2-F | GCAGGACTGGAAGCCATCAT |
| M-NPAS2-R | GAGGGGCTAGGCACATTGTT |
| M-H3F3B-F | GTGCCCTTCAGGAGGCTA |
| M-H3F3B-R | CGTGGATGGCACACAGATTG |
| M-SCML1-F | TGCAGCCCCCTCTAGGATTT |
| M-SCML1-R | GGCTGATGCTCCAAGACAGT |
| M-INSIG1-F | AGCGTTATGCGCTGTATTGC |
| M-INSIG1-R | TTTTCCGGAACACCCATAGC |
| M-Bmal1-F | AAAAGAGGCGTCGGGACAAA |
| M-Bmal1-R | AAATAGCTGTCGCCCTCTGA |
| M-cry-F | CAGCAGATAACAGACGCCCT |
| M-cry-R | GACGAAGCCGTCAATCTCCT |
| M-Rev-erb-F | TCGGAGGAGCATTCAGCAAA |
| M-Rev-erb-R | GCTCATAGGACACACCAGATG |
| M-per2-F | AGCTGACGCACACAAAGAAC |
| M-per2-R | GAGGGATTCTAGGCGCTTCAT |
| M-RORa-F | TGAAGGCTGCAAGGGCTTTT |
| M-RORa-R | AAACACCACCTCTAGCGAGC |
